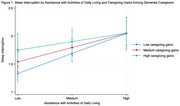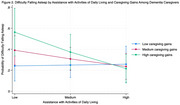# Caregiving stressors and sleep outcomes among dementia caregivers: examining caregiving gains as a moderator

**DOI:** 10.1002/alz70858_099915

**Published:** 2025-12-25

**Authors:** Fei Wang, Meng Huo

**Affiliations:** ^1^ The University of Tennessee Knoxville, Knoxville, TN, USA; ^2^ UC Davis, Davis, CA, USA

## Abstract

**Background:**

In the United States, approximately 67% of caregivers of older adults with dementia experience sleep problems. Yet, we know surprisingly little about ways to protect against the negative impact of dementia caregiving on sleep. Building on the documented health benefits of coping resources such as perceived gains from caregiving (i.e., caregiving gains), this study examined the moderating effects of caregiving gains on the associations between caregiving stressors and sleep.

**Method:**

Data were drawn from the 2017 *National Study of Caregiving* and *National Health and Aging Trends Study*. Participants included 894 caregivers (*M_age_* = 61.77 years old) of Medicare enrollees aged 65 and older with dementia in the U.S. Caregivers reported their objective (i.e., ADL assistance, IADL assistance) and subjective (i.e., caregiver role overload) stressors. Sleep outcomes included sleep interruption, sleep satisfaction, any difficulty falling asleep, and waking up at night. We estimated multiple linear regressions and logistic regressions, adjusting for clustering and applying population weight.

**Result:**

ADL assistance was positively associated with sleep interruption, but this association was weaker among caregivers with higher caregiving gains than those with lower caregiving gains (Figure 1). ADL assistance was not associated with the likelihood of experiencing difficulty falling asleep. However, when caregivers experienced higher caregiving gains, more ADL assistance was associated with a reduced likelihood of experiencing difficulty falling asleep (Figure 2). It is worth noting that overall, caregivers with higher caregiving gains experienced more sleep problems than those with lower caregiving gains (Figures 1&2). Role overload was positively associated with sleep interruption but this association was not moderated by caregiving gains.

**Conclusion:**

The findings identified the moderating role of caregiving gains in the association between an objective caregiver stressor (i.e., ADL assistance) and sleep, offering a nuanced understanding of the impact of caregiving gains. That is, the health benefits of caregiving gains may be conditional for dementia caregivers. Further research is needed to explore the meaning and implications of caregiving gains in this population. This line of inquiry will help inform interventions focused on empowering caregivers to recognize the positive aspects of caregiving and promoting caregivers’ health outcomes.